# Diversity in chemical subunits and linkages: a key molecular determinant of microbial richness, microbiota interactions, and substrate utilization

**DOI:** 10.1128/spectrum.02618-24

**Published:** 2025-03-06

**Authors:** Hugh C. McCullough, Hyun-Seob Song, Jennifer M. Auchtung

**Affiliations:** 1 Department of Food Science and Technology, University of Nebraska-Lincoln 14719 , Lincoln, Nebraska, USA; 2 Nebraska Food for Health Center, University of Nebraska-Lincoln 14719 , Lincoln, Nebraska, USA; 3 Department of Biological Systems Engineering, University of Nebraska-Lincoln 14719 , Lincoln, Nebraska, USA; Lerner Research Institute, Cleveland, Ohio, USA

**Keywords:** dietary carbohydrates, niche, microbial interactions, microbial richness

## Abstract

Dietary fibers play a significant role in shaping the composition and function of microbial communities in the human colon. Our understanding of the specific chemical traits of dietary fibers that influence microbial diversity, interactions, and function remains limited. Toward filling this knowledge gap, we developed a novel measure, termed Chemical Subunits and Linkages (CheSL) Shannon diversity, to characterize the effects of carbohydrate complexity on human fecal bacteria cultured *in vitro* under controlled, continuous flow conditions using media that systematically varied in carbohydrate composition. Our analysis revealed that CheSL Shannon diversity demonstrated a strong Pearson correlation with microbial richness across multiple fecal samples and study designs. Additionally, we observed that microbial communities in media with higher CheSL Shannon diversity scores exhibited greater peptide utilization and more connected, reproducible structures in computationally inferred microbial interaction networks. Taken together, these findings demonstrate that CheSL Shannon diversity can be a useful tool to quantify the effects of carbohydrate complexity on microbial diversity, metabolic potential, and interactions. Furthermore, our work highlights how robust and stable community data can be generated by engineering media composition and structure. These studies provide a valuable framework for future research on microbial community interactions and their potential impacts on host health.

**IMPORTANCE:**

For the human adult gut microbiota, higher microbial diversity strongly correlates with positive health outcomes. This correlation is likely due to increased community resilience that results from functional redundancy that can occur within diverse communities. While previous studies have shown that dietary fibers influence microbiota composition and function, we lack a complete mechanistic understanding of how differences in the composition of fibers are likely to functionally impact microbiota diversity. To address this need, we developed Chemical Subunits and Linkages Shannon diversity, a novel measure that describes carbohydrate complexity. Using this measure, we were able to correlate changes in carbohydrate complexity with alterations in microbial diversity and interspecies interactions. Overall, these analyses provide new perspectives on dietary optimization strategies to improve human health.

## INTRODUCTION

Members of the gut microbiota are known to contribute to different aspects of human health, including metabolizing otherwise inaccessible dietary substrates, educating the immune system, and providing protection from pathogen infection (1–4). Ongoing research efforts focus on developing treatments and interventions to engineering the gut microbiota to improve host health (5–8); approaches have included an addition of new dietary substrates (e.g., prebiotics and dietary fibers [9–11]), new microbes (e.g., probiotics and live microbial therapeutics [1, 12, 13]), or combinations of substrates and microbes (e.g., synbiotics [2, 14]). Approaches to engineering the microbiota will continue to benefit from improved understanding of how ecological interactions govern microbiota assembly, composition, and function. Previous studies have shown diets are a major driver of microbial composition (15). In mice, the consumption of a westernized diet caused large changes in microbiota composition compared to consumption of a low-fat, low-sugar diet enriched in complex polysaccharides (16, 17). Similarly, long-term dietary patterns and microbiota composition are correlated in humans (18). Dietary fibers are of particular interest for gut microbiota modulation because they cannot be metabolized by human enzymes, and gut microbiota metabolism of these substrates can produce products (e.g., butyrate) metabolized by the host (19–22).

The “nutrient-niche theory” proposed by Freter et al. (23, 24) can serve as a basic framework to understand the impacts of diets on microbiota composition. It states that the metabolic potential of microbial community members and the availability of nutrients dictate how niches are occupied (23, 24). Particularly, increasing the number of unique utilizable substrates expands the fundamental niche space potentially facilitating increased microbial richness. However, competition for limiting nutrients and environmental conditions that limit growth may restrict the ability of microbes to colonize (25). Recent work has begun to investigate how carbohydrate structural diversity impacts microbial community composition. Chung et al. used continuous flow bioreactors to demonstrate that more complex fibers (higher levels of branching, monosaccharide diversity) and/or mixtures of fibers increased microbial community richness and diversity compared to simple fibers, although there was some variation between the two fecal communities tested (26). Yao et al. observed similar effects culturing human fecal microbiotas in batch cultures passaged under high dilution pressure, with the more complex fiber sorghum arabinoxylan supporting higher microbial community richness and diversity than the simple fiber inulin or mixtures of the primary monosaccharides found in either fiber (27). However, a follow-up study looking at two different complex carbohydrates, white sorghum arabinoxylan and red sorghum arabinoxylan, observed that the subtle differences in carbohydrate structure could have significant impacts on the microbial richness of fecal communities cultured under high dilution pressure (28). Using a defined community of 10 fecal microbes, Ostrem Loss et al. demonstrated that higher carbohydrate complexity and low sugar concentrations led to higher community diversity by increasing the ratio of positive to negative interactions between strains (29).

**Fig 1:**
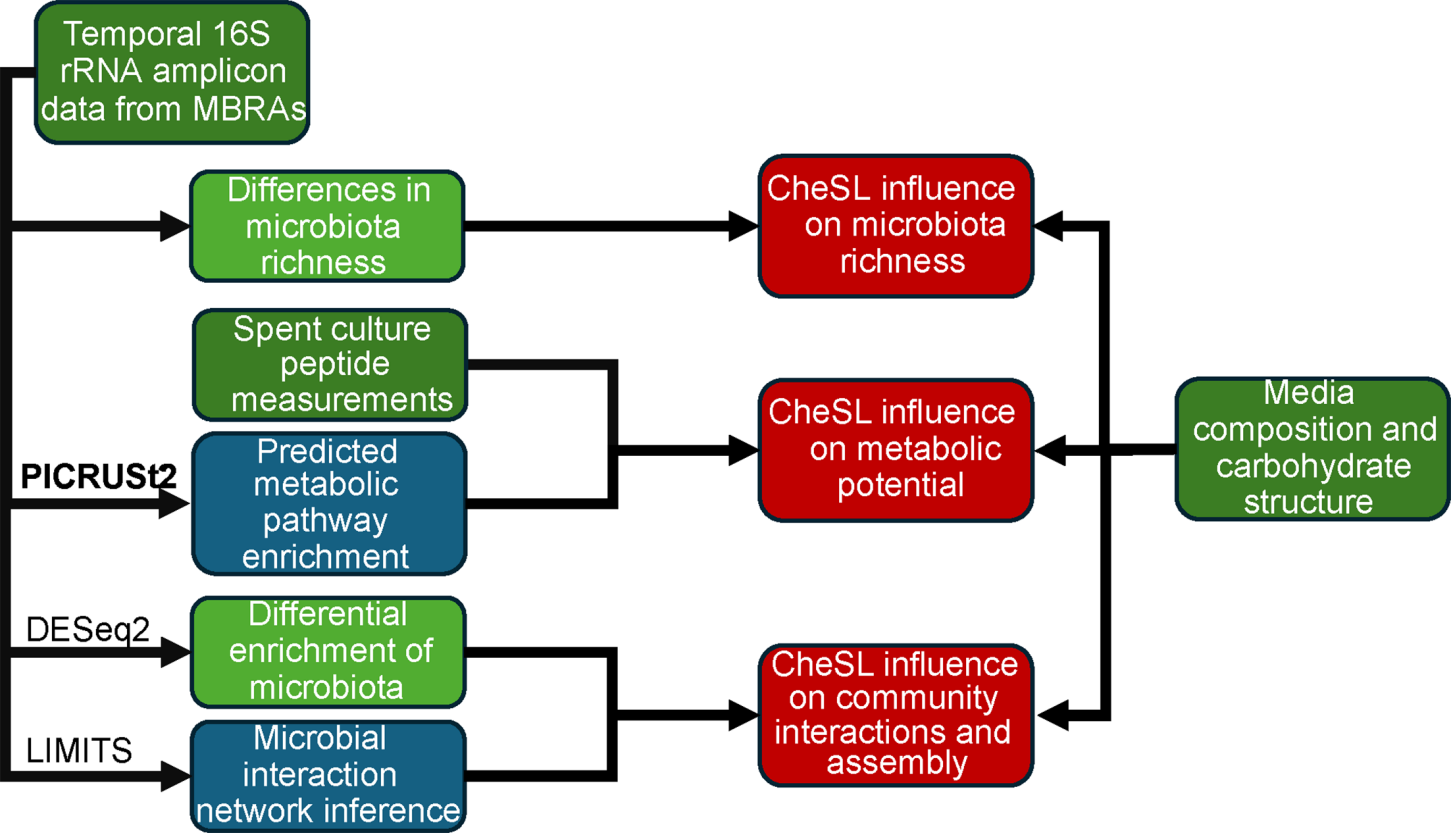
Graphical abstract. A conceptual overview of this study, centered on applicability of CheSL metrics to different community culture traits. Empirical data are shown in dark green, post-analysis traits are shown in light green, inferred characteristics are shown in blue, and the interpretations are shown in red.

In this study, we analyzed the response of human fecal microbial communities to changes in the diversity and concentration of carbohydrates through a combination of *in vitro* experiments and computational modeling using a previously described continuous flow model of the lumen of the distal colon (30). Our workflow was composed of three parts (Fig. 1): (i) data generation and analysis to identify key traits of carbohydrates determining microbial richness, (ii) post-analysis of metabolic potential and activity, and (iii) network inference to predict community structure-microbial interactions and central microbiota. Inspired by the nutrient-niche theory, we developed a molecular-level diversity metric of carbohydrates termed Chemical Subunits and Linkages (CheSL) Shannon diversity, which we used to quantify carbohydrate diversity for mixtures of carbohydrates based on their internal linkages and subunits. Communities cultured in media with high CheSL Shannon diversity demonstrated strong positive correlations with microbial richness, peptide utilization (measured and predicted through Stickland fermentation), and microbial interactions in computationally inferred networks. Through these combined data analyses and model predictions, we also determined that microbial communities assembled under conditions with higher CheSL Shannon diversity led to more reproducible interactions with conserved central taxa (*Bacteroides*, *Lachnospiraceae*, and *Ruminococcaceae*). Finally, we were able to draw parallels between the enrichment of specific microbes in the presence of carbohydrate mixtures that were consistent with predicted abundances of metabolic pathways and enrichment previously observed *in vivo*.

## RESULTS

### Developing metrics to quantify carbohydrate complexity

To investigate the potential contribution of carbohydrate complexity to unique niches within a medium (Table S1), we developed a new metric termed CheSL, which enables quantifying carbohydrate complexity based on the number of unique free monosaccharides and linkages present in each carbohydrate. Accounting for internal linkages in evaluating carbohydrate complexity is critical because polysaccharides may provide one or more unique niches depending on their complexity, and the likelihood that enzymes required for degradation are encoded within a single microbe may vary based on linkage complexity. For example, previous studies have demonstrated that while *Bifidobacterium longum* (31) and *Bacteroides thetaiotaomicron*, two common members of the gut microbiota, possess enzymes necessary for the complete degradation of the simple fiber starch (32), neither *B. longum* nor *B. caccae* can fully metabolize arabinogalactan, a more complex polysaccharide composed of five different types of glycosidic bonds that connect primarily arabinose and galactose monomers (Table 1) (33).

**TABLE 1:** Linkages of disaccharides and polysaccharides tested.^*a*^

Polymeric carbohydrate	Linkage	Linkage abundance	Monosaccharide	Reference
Arabinogalactan	Gal tp	0.257	Galactose	Goellner et al. (34)
	Gal 3p	0.049	Galactose	
	Gal 6p	0.258	Galactose	
	Gal 3,4p	0.01	Galactose	
	Gal 3,6p	0.289	Galactose	
	Ara tf	0.045	Arabinose	
	Ara tp	0.048	Arabinose	
	Ara 3f	0.044	Arabinose	
Starch—amylose (25%)	α-(l-4)	0.25	Glucose	Ai and Jane (35)
Starch—amylopectin (75%)	α-(l-4)	0.7125	Glucose	Ai and Jane (35)
	α-(l-6)	0.0375	Glucose	
Inulin	β(2—1)	.862	Fructose	Yao et al. (27)
	α-(l-2)	.138	Glucose	
Cellobiose	β (1-4)	1	Glucose	French (36)
Maltose	α-(l-4)	1	Glucose	Nawaz et al. (37)
Fructans	Fru tf	0.005	Fructose	King et al. (38)
	Fru β(2-1)f	0.045	Fructose	
	Fru β (2-6)f	0.34	Fructose	
	Fru β (1-6)f	0.22	Fructose	
	Glc tp	0.05	Glucose	
	Glc (l-6)p	0.34	Glucose	

^*a*^ Linkages from the different polysaccharides used in this study, with estimated linkage abundances from literature, and in the case of starch, fractions of amylose and amylopectin determined as described in Materials and Methods. For linkages, t indicates terminal, 3,4,6, refers to which carbon the bond is on, f indicates it is in the furanose form and p, the pyranose form.

The primary inputs for CheSL include the fractional composition of linkages in each carbohydrate and the fractional composition of carbohydrates in each medium. This information can be effectively represented by defining a linkage matrix (**L**) and media matrix (**M**), respectively.

The matrix **L** representing the fractional composition of the linkage arrangements present across the carbohydrates contained in all media can be given as follows:


(1) $$L=\left[\begin{array}{ccc} l_{1,1} & \cdots & l_{1,n_{C}}\\ \vdots & \ddots & \vdots \\ l_{n_{L},1} & \cdots & l_{n_{L},n_{C}} \end{array}\right]$$

where the rows and columns in **L** represent linkage arrangements and carbohydrates, respectively; $n_{L}$ denotes the total number of unique linkages associated with subunits contained in all carbohydrates; the ${\left(i,j\right)}^{tℎ}$ element of **L**, that is, $l_{i,j}$, denotes the proportion of linkage arrangement *i* within carbohydrate *j*.

The matrix **M** representing media-specific carbohydrate composition can be given as follows:


(2) $$M=\left[\begin{array}{ccc} m_{1,1} & \cdots & m_{1,n_{M}}\\ \vdots & \ddots & \vdots \\ m_{n_{C},1} & \cdots & m_{n_{C},n_{M}} \end{array}\right]$$

where the rows and columns in **M** represent carbohydrates and media, respectively; $n_{c}$ and $n_{M}$ denote the total numbers of unique carbohydrates and media used in experiments, respectively; the ${\left(j,k\right)}^{tℎ}$ element of **M**, that is, $m_{j,k}$, denotes the concentration of a specific carbohydrate *j* in a medium *k*.


(3) $$L=\left[\begin{array}{ccc} l_{1,1} & \cdots & l_{1,n_{C}}\\ \vdots & \ddots & \vdots \\ l_{n_{L},1} & \cdots & l_{n_{L},n_{C}} \end{array}\right]$$

We can also define a $\left(n_{L}\times n_{M}\right)$ matrix **S** to represent the concentrations of linkage arrangements (or subunits) in media, which are readily obtainable by multiplying **L** and **M**, that is,


(4) S=LM

where the rows and columns in **S** represent linkage arrangements and media, respectively; the ${\left(i,k\right)}^{tℎ}$ element of **S**, that is, $s_{i,k}$, denotes the *i*th linkage arrangement (or free subunit) concentration for the *k*th medium. The fractional abundance of the $i^{tℎ}$ linkage arrangement (or free subunit) within the $k^{tℎ}$ medium, denoted by $f_{i,k}$, is simply calculated from $s_{i,k}(i=1,2,\ldots ,n_{L})$ as follows:


(5) $$f_{i,k}=\;\frac{s_{i,k}}{\sum_{i^{\prime }=1}^{n_{L}}s_{i^{\prime },k}}$$

We can finally calculate CheSL Shannon diversity and CheSL Shannon evenness for the *k*th medium, denoted CSI*_k_* and CSE*_k_*, respectively, using the equations below:


(6) $$CSI_{k}=-\sum_{i\in I_{NZ,k}}f_{i,k}\ln\left(f_{i,k}\right)$$

(7) $$CSE_{k}=\;\frac{CSI_{k}}{\ln\left(|I_{NZ,k}|\right)}$$

where $I_{NZ,k}$ denotes the indices of non-zero elements of $s_{k}$. For CheSL Shannon evenness, we divide $CSI_{k}$ by the logarithm of the number of non-zero elements in $s_{k}$ (or logarithm of the cardinality of $I_{NZ,k}$), which represents the logarithm of CheSL richness.

In the metric we developed, monomeric glucose was assigned a CheSL value of 0, because pathways to import and metabolize glucose are conserved across many species of bacteria (39–43) and as a result, glucose metabolism was unlikely to provide a unique niche for microbes within most communities. Other unique monosaccharides (e.g., arabinose, N-acetylglucosamine, and d-glucuronic acid) were assigned CheSL richness values of one, as pathways for their uptake and utilization were not expected to be as widely conserved as glucose and could represent unique niches. Other CheSL values for polysaccharides were primarily assigned based on the number of unique linkage arrangements, as the capability to metabolize monosaccharides liberated from polysaccharides was expected to be part of the same unique niche. With the exception of soluble starch, carbohydrate composition and linkage data were estimated based on previously published literature (Table 1).

**Fig 2:**
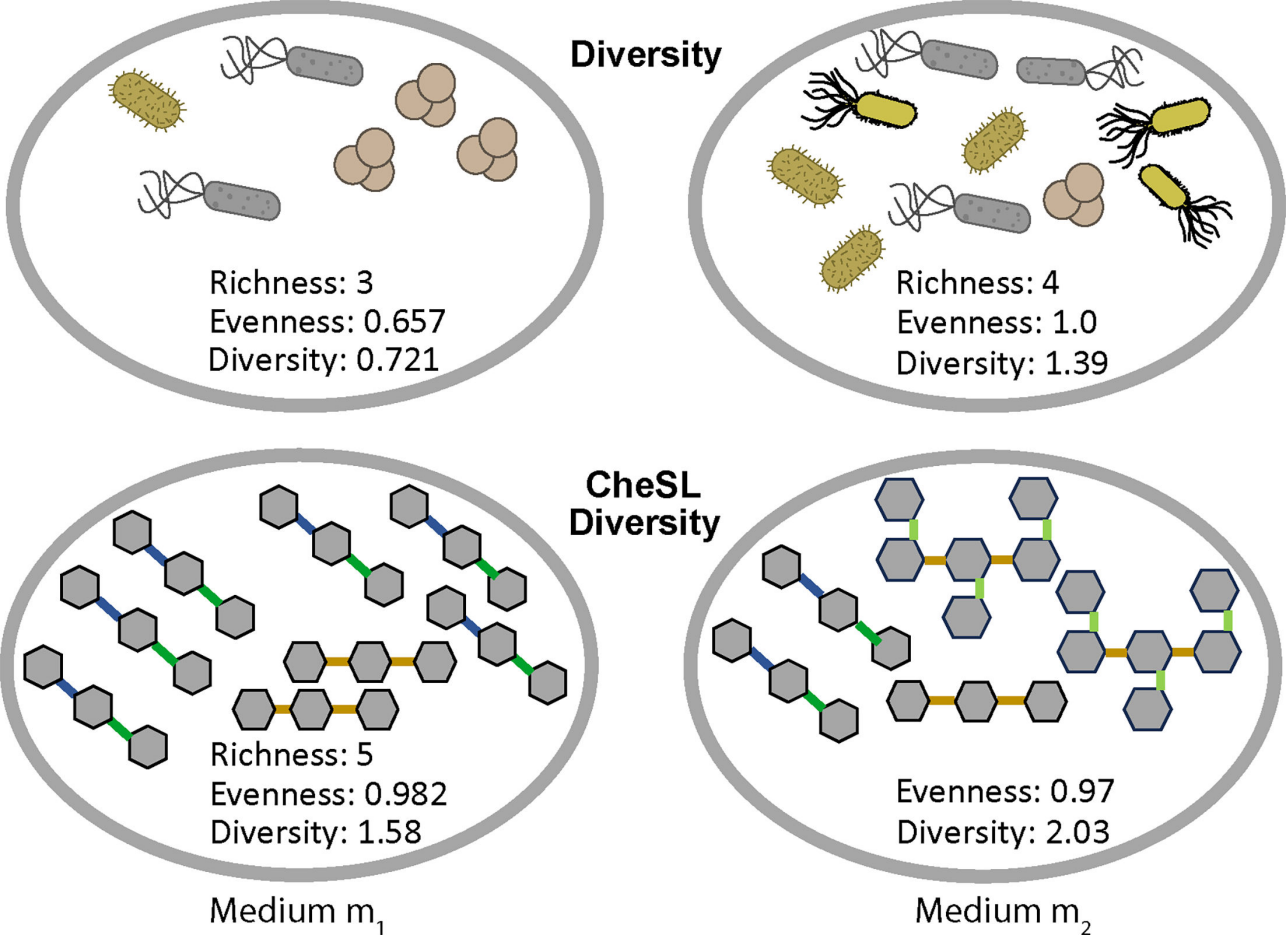
Graphical representation of Carbohydrate Chemical Subunits and Linkages (CheSL) metrics. Graphical representations of approaches to calculate microbial and CheSL richness, Shannon evenness, and Shannon diversity. In these examples, different microbes are represented by different shapes and colors, and different linkages are color-coded.

Using the two hypothetical media exemplified at the bottom of Fig. 2, we illustrate how to calculate CheSL richness, CheSL Shannon diversity, and CheSL evenness metrics. Across these two media, there are three carbohydrates. Carbohydrate 1 has two linkage arrangements, of which 66% are arrangement 1 and 33% are arrangement 2. Carbohydrate 2 was a polysaccharide, with three linkage arrangements (3, 4, and 5) in equal proportions. Carbohydrate 3 contains three linkage arrangements, 50% are linkage arrangement 6, 33.33% are linkage arrangement 7, and 16.66% are linkage arrangement 8. The linkage matrix **L** is below:


(8) $$L=\;\left[\begin{array}{ccc} .6666 & 0 & 0\\ .3333 & 0 & 0\\ 0 & .3333 & 0\\ 0 & .3333 & 0\\ 0 & .3333 & 0\\ 0 & 0 & .5\\ 0 & 0 & .3333\\ 0 & 0 & .1666 \end{array}\right]$$

The first medium in the study, medium 1, contains 1 g/L carbohydrate 1 and 2 g/L carbohydrate 2. The second medium in the study, , contains 1 g/L carbohydrate 1, 2 g/L carbohydrate 2, and 2 g/L carbohydrate 3. The media matrix (**M**) is shown below:


(9) $$M=\;\left[\begin{array}{cc} 1 & 1\\ 2 & 2\\ 0 & 2 \end{array}\right]$$

The concentration of linkages, **S**, can be calculated from **L** and **M**.


$$\;\;S=LM=\left[\begin{array}{ccc} .6666 & 0 & 0\\ .3333 & 0 & 0\\ 0 & .3333 & 0\\ 0 & .3333 & 0\\ 0 & .3333 & 0\\ 0 & 0 & .5\\ 0 & 0 & .3333\\ 0 & 0 & .1666 \end{array}\right]\left[\begin{array}{cc} 1 & 1\\ 2 & 2\\ 0 & 2 \end{array}\right]=\;\left[\begin{array}{cc} .6666 & .6666\\ .3333 & .3333\\ .6666 & .6666\\ .6666 & .6666\\ .6666 & .6666\\ 0 & 1\\ 0 & .6666\\ 0 & .3333 \end{array}\right]$$

(10)





These calculations demonstrate that the medium 1, has 0.3333 g/L worth of carbohydrate associated with linkage 2 and 0.6666 g/L associated with linkage arrangements 1, 3, 4, and 5. Medium 2 has 0.6666 g/L worth of carbohydrate associated with linkage arrangements 1, 3, 4, 5, and 7, 0.3333 g/L worth of carbohydrate associated with linkage arrangements 2 and 8, and 1 g/L worth of carbohydrate associated with linkage arrangement 6.

From this, we calculated the fractional contributions of each linkage pool to the total carbohydrate concentration in each media as matrix **F**. For *f*_*i,1*_, this was 0.222 for linkage arrangements 1, 3, 4, and 5, and 0.111 for linkage arrangement 2. For *f*_*i,2*_, this was 0.1333 for linkages 1, 3, 4, 5, 7, 0.0666 for linkage arrangements 2 and 8, and 0.2 for linkage arrangement 6.


(10) $$F=\left[\begin{array}{cc} .2222 & .1333\\ .1111 & .0666\\ .2222 & .1333\\ .2222 & .1333\\ .2222 & .1333\\ 0 & .2\\ 0 & .1333\\ 0 & .0666 \end{array}\right]$$

Using this fractional matrix and the formulas described above, we determined that for medium 1**,** the CheSL richness was 5, CheSL Shannon diversity was 1.58, and CheSL Shannon evenness was 0.982, whereas for medium 2, the CheSL richness was 8, CheSL Shannon diversity was 2.03, and CheSL Shannon evenness was 0.97.

### CheSL Shannon diversity connects carbohydrate complexity to microbial richness

We hypothesized that increasing the number of unique utilizable substrates would expand the fundamental niche space and facilitate increased microbial richness. To test this, microbial communities from the fecal samples of two healthy humans (Fecal Samples A and B [FSA and FSB]) were cultured in continuous-flow minibioreactor arrays (MBRAs) with media that differed in composition and abundance of carbohydrates. Previously, we demonstrated that community assembly in MBRAs was a result of environmental filtering that was selected for core, cultivatable taxa under culture conditions, as well as stochastic processes that contributed to variation between replicate communities (30). We expected that unique utilizable carbohydrate linkages would contribute to community assembly primarily through environmental filtering, but that stochastic processes, such as dispersal limitation from fecal samples and stochastic birth/death, would also impact assembly.

Replicate cultures were inoculated into four media (NS, MM, 5MM, and IL) that varied in composition and abundance of carbohydrates but were composed of the same base of proteins, lipids, and trace minerals (Table S1). All media contained low levels of inulin and arabinogalactan. NS also contained low levels of mono (glucose) and disaccharides (cellobiose and maltose) and had CheSL richness, Shannon diversity, and Shannon evenness measures of 12, 1.8, and 0.71, respectively. MM contained low levels of mucosal monosaccharides and soluble starch and had CheSL richness, Shannon diversity, and Shannon evenness measures of 16, 1.9, and 0.70, respectively. 5 MM contained higher levels of mucosal monosaccharides and soluble starch compared to MM and had CheSL richness, Shannon diversity, and Shannon evenness measures of 16, 1.5, and 0.56, respectively. IL was supplemented with ileostomy effluent, which likely contains a complex mixture of carbohydrates and other factors, requiring approximation of substrate concentrations from available literature (44, 45); CheSL richness, Shannon diversity, and Shannon evenness measures were estimated at 24, 2.0, and 0.62, respectively. Samples were collected from communities daily, and changes in microbial community composition were determined through sequencing the V4 region of the 16S rRNA gene. While data were generated from both fecal samples cultured in NS, MM, and IL, contamination limited the collection of data from 5 MM to FSB only.

**Fig 3:**
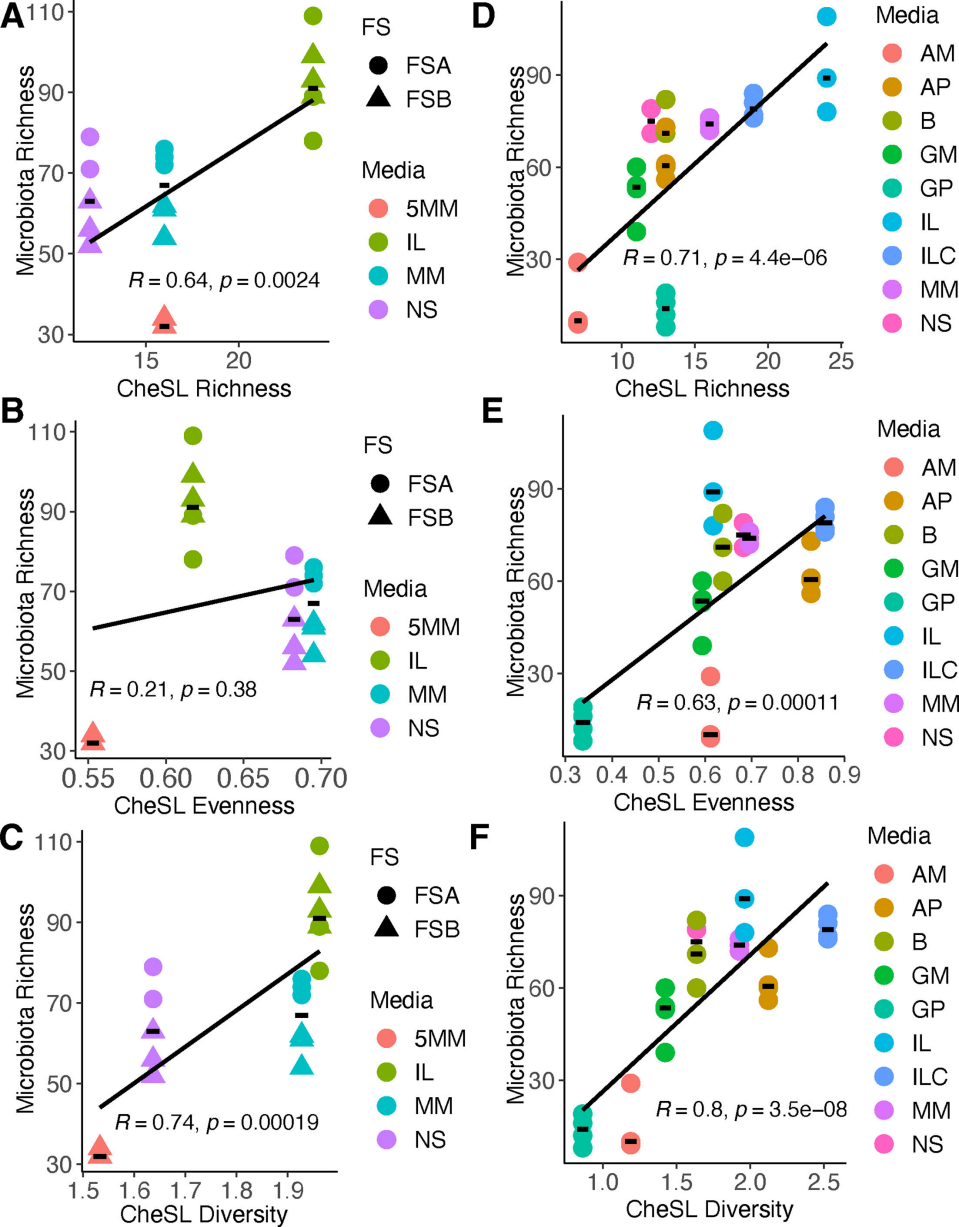
Effects of CheSL richness, CheSL Shannon evenness, and CheSL Shannon diversity on microbiota richness. Correlations of shared microbiota richness (observed ASVs shared across a time window as described in Materials and Methods) with (**A**) CheSL richness, (**B**) CheSL Shannon evenness, and (**C**) CheSL Shannon diversity for FSA (circles) and FSB (diamonds). Correlations of shared microbiota richness with (**D**) CheSL richness, (**E**) CheSL Shannon evenness, and (**F**) CheSL Shannon diversity for FSA communities cultured in additional media along with those described in panels (**A**)–(**C**). In all plots, Pearson correlation coefficients and *P* values are reported. Correlation analysis for FSA and FSB alone in the media shown in panels (**A**)–(**C**) and for FSA in the new media shown in panels (**D**)–(**F**) can be found in Fig. S1.

Contrary to our original expectation, we found that microbiota richness (number of observed ASVs) was most strongly correlated with CheSL Shannon diversity rather than CheSL richness (Fig. 3). Specifically, we observed a Pearson correlation coefficient between ASV richness and CheSL richness of *R* = 0.64 (*P* = 0.0024, Fig. 3A), between ASV richness and CheSL Shannon evenness of 0.21 (*P* = 0.38, Fig. 3B), and between ASV richness and CheSL Shannon diversity of *R* = 0.74 (*P* = 0.00019, Fig. 3C). Individual analysis of each fecal sample (Fig. S1A through F), clearly indicated that the strong correlation was primarily a result of observations with FSB, as correlations for FSA were lower. One factor potentially contributing to this observation could be the relatively narrow range of CheSL Shannon diversity and CheSL Shannon evenness tested for FSA.

We evaluated the effects on microbial richness when FSA was cultured in additional media that varied across a broader range of CheSL Shannon diversity and CheSL evenness (Table 2). Unfortunately, FSB samples were no longer available and could not be used for further testing. Medium B was similar to NS, but also had low levels of soluble starch; CheSL richness, CHeSL Shannon diversity, and CheSL Shannon evenness measures were 13, 1.64, and 0.63, respectively. ILC included a mixture of carbohydrates inferred to be present in IL at low concentrations (44, 45); CheSL richness, CheSL Shannon diversity, and CheSL Shannon evenness measures were 19, 2.5, and 0.86, respectively. The remaining four media had a single carbohydrate polymer or its monomers present at higher levels, but this carbohydrate varied in complexity. GP medium had higher concentrations of glucose polysaccharide (starch) with CheSL richness, CheSL Shannon diversity, and CheSL Shannon evenness measures of 13, 0.86, and 0.34, respectively. GM medium had higher concentrations of glucose monosaccharide, with CheSL richness, CheSL Shannon diversity, and CheSL Shannon evenness measures of 11, 1.4, and 0.59, respectively. AP medium had higher concentrations of arabinogalactan polysaccharide, with CheSL richness, CheSL Shannon diversity, and CheSL Shannon evenness measures of 13, 2.1, and 0.82, respectively. Finally, AM medium had high concentrations of the arabinogalactan monomers galactose and arabinose, with CheSL richness, CheSL Shannon diversity, and CheSL Shannon evenness measures of 7, 1.2, and 0.61, respectively. We cultured replicate communities from FSA in these different media in continuous-flow MBRAs and analyzed changes in microbial community composition through sequencing of the V4 region of the 16S rRNA gene.

**TABLE 2:** Variations in bioreactor medium

Carbohydrate (g/L)	B^*a*^	ILC^*a*^	GP^*a*^	GM^*a*^	AP^*a*^	AM^*a*^
Arabinogalactan	0.1	0.05	0.1	0.1	2.0	-^*b*^
Inulin	0.2	0.05	0.2	0.2	0.2	0.2
Soluble starch	0.2	0.05	2.0	0.2	0.2	0.2
Cellobiose	0.15	0.05	0.15	0.15	0.15	0.15
Maltose	0.15	-	0.15	0.15	0.15	0.15
Glucose	0.04	-	0.04	2.0	0.04	0.04
Fructose	-	0.05	-	-	-	-
Galactose	-	0.05	-	-	-	1.71
Arabinose	-	-	-	-	-	0.29
N-acetylneuraminic acid	-	0.05	-	-	-	-
N-acetylglucosamine	-	0.05	-	-	-	-
l-fucose	-	0.05	-	-	-	-
d-glucuronic acid	-	0.05	-	-	-	-
Total carbohydrate (g/L)	0.84	0.5	2.64	2.8	2.74	2.74

^*a*^ Abbreviations for basal media variations: B: basal medium; ILC: ileostomy carbohydrates; GP: glucose polymer (soluble starch); GM: glucose monomer; AP: arabinogalactan polymer; AM: arabinogalactan monomers.

^*b*^ “-” indicates absence of indicated carbohydrate.

Adding this new data to our existing FSA data, we observed higher correlations between ASV richness and CheSL richness (Fig. 3D; *R* = 0.71, *P* = 4.4 × 10^−6^), CheSL Shannon evenness (Fig. 3E, *R* = 0.63, *P* = 0.00011), and CheSL Shannon diversity (Fig. 3F, *R* = 0.8, *P* = 3.5 × 10^−8^) across FSA samples. Analysis of FSA samples from this second experiment alone yielded higher correlations (Fig. S1I), more consistent with what was observed for FSB cultured across a range of media (Fig. S1C). As the two FSA experiments were performed on a cryopreserved fecal sample that had been stored for several years in between experiments, the high levels of correlation across the two experiments indicated the overall robustness of the observations.

To evaluate the potential broader applicability of CheSL richness, CheSL Shannon diversity, and CheSL Shannon evenness, we also evaluated two previously published data sets examining the effects of carbohydrate diversity on fecal microbial community assembly (26, 27). For these data sets, carbohydrate linkage information was estimated from the available literature (Table S2), and microbial richness data were obtained from the text and/or supplementary information (26–28, 46–48). We observed similar correlations between microbiota richness and CheSL richness, CheSL Shannon evenness, and CheSL Shannon diversity (Fig. S2), across these studies, although substrate complexity and the needed approximations made for certain polysaccharides used (i.e., pectin), may have reduced the statistical significance of data analyzed from Chung et al. (26). Overall, these analyses demonstrated that CheSL Shannon diversity can be broadly applied, but also highlighted potential limitations for use with certain fecal communities and carbohydrate structures.

### CheSL Shannon diversity predicts peptide utilization

**Fig 4:**
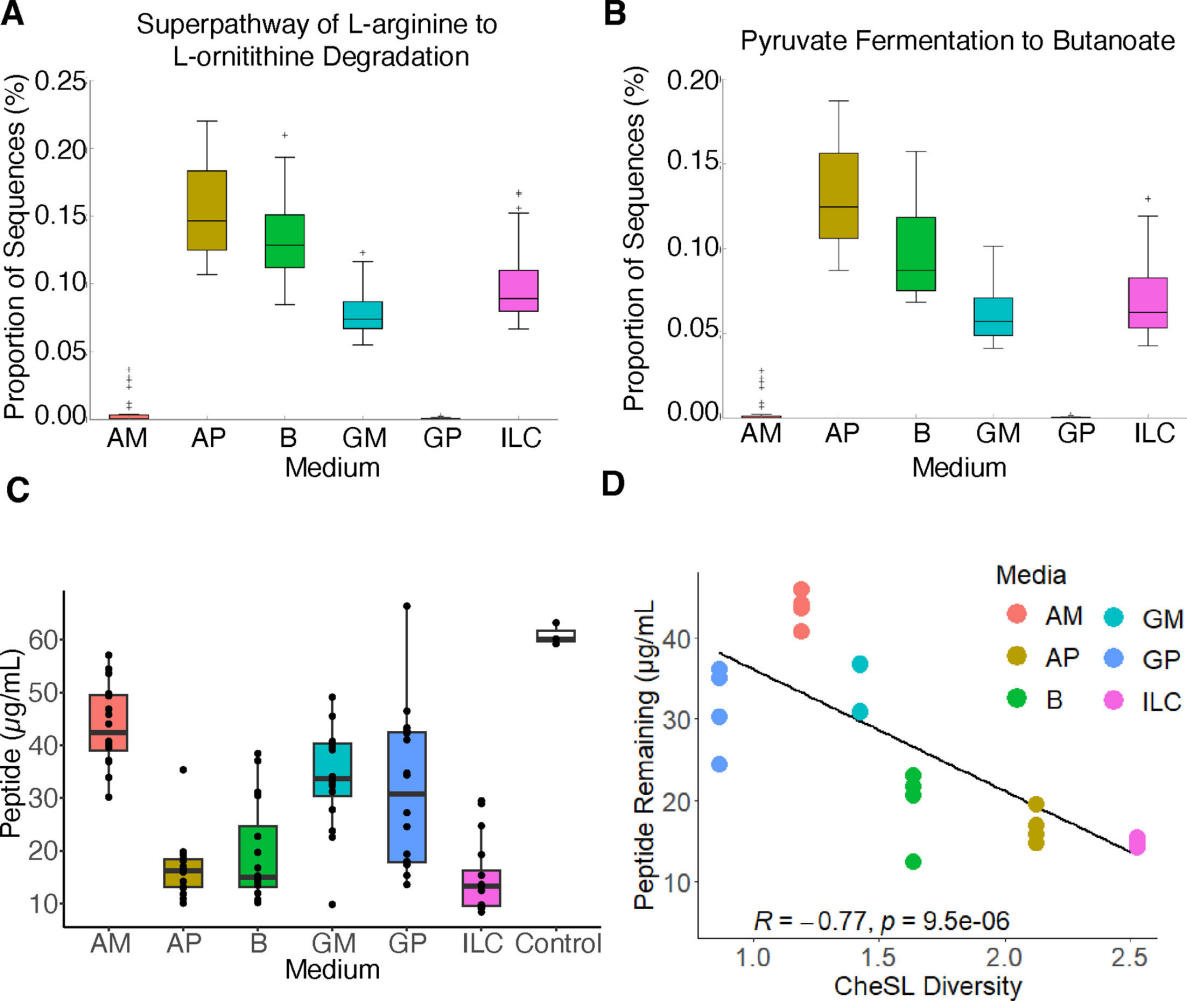
Differentially abundant functional pathways suggest divergent metabolism and production of beneficial compounds. (**A and B**) STAMP generated plots of pathways differentially abundant in FSA communities cultured in media with high CheSL Shannon diversity complexity and low CheSL Shannon diversity. (**C**) Total peptide concentrations measured in steady-state culture of each media type exhibiting differences in peptide utilization by communities compared to fresh media control. (**D**) Correlation between CheSL Shannon diversity and the peptide concentration in spent medium. Pearson correlation coefficient and *P* value are reported. Additional correlations can be found in Fig. S3.

To investigate potential functional differences between communities that may be drivers of community assembly or function, we used PICRUSt2 to infer genome content from 16S rRNA gene sequences and STAMP to identify differentially abundant functional pathways between communities cultured in different media. We focused on data from FSA cultured in the media described in Table 2 because we also had access to cell-free culture supernatants for further testing. One observation of interest, given our recent studies indicating the contributions of Stickland fermentation to persistence of *Clostridioides difficile* in fecal communities cultured in MBRA with NS medium (49), was enrichment for taxa potentially encoding genes for l-arginine degradation (Stickland fermentation; Fig. 4A) in microbial communities with higher shared richness (cultured in B, GM, AP, and ILC) and lower in microbial communities with lower shared richness (cultured in AM and GP). We also observed the predicted pathways for fermentation of pyruvate to butanoate (butyrate; Fig. 4B) were enriched in these same microbial communities. We hypothesized that the differential abundance of Stickland fermentation pathways could indicate differences in peptide fermentation between communities. Using a commercial assay to measure levels of peptide remaining in spent culture supernatants, we found that peptide utilization varied between communities cultured in different types of media (Fig. 4C). When we performed a correlation between peptide utilization and CheSL Shannon diversity, we observed a strong correlation (*R* = −0.77, *P* = 9.5 × 10^−6^, Fig. 4D), similar to that observed between peptide utilization and microbiota richness alone (Fig. S3A) and higher than that observed for CheSL Shannon evenness (Fig. S3B). From this data, we infer that for communities cultured in the presence of more complex mixtures of carbohydrates, peptide utilization may serve as an additional substrate niche dependent on community composition; alternatively, these communities may have higher needs for peptide utilization to facilitate carbohydrate degradation capabilities.

### Bacteroides and Lachnospiraceae are central members in conserved, rich communities

As microbial interactions can have significant impacts on microbial community assembly, stability, and function (25), we were interested in investigating how variations in carbohydrate composition affected microbial interactions. Thus, we used LIMITS, a computational algorithm that infers microbial interactions by fitting a discrete Lotka-Volterra model against the temporal profiles of ASV relative abundance data (50, 51), to analyze our sequence data. The LIMITS equation was derived by implicitly assuming that total community biomass does not significantly vary in time. Thus, performance is improved when LIMITS is conducted with data collected from communities with minimal variations of the total biomass, such as those found when culturing microbial communities under continuous-flow conditions approaching steady state. However, LIMITS ignores temporal variations of interspecies interactions by assuming interaction coefficients to be constant in time. While relevant in certain cases where microbial interactions are expected not to significantly change, this assumption is invalid in general (52, 53). To address this limitation, we employed a moving window approach by: (i) dividing the data into multiple subsets, each representing a shorter time period; (ii) using LIMITS to identify (constant) microbial interactions within these shorter periods; and (iii) iteratively inferring the network by shifting the time window to subsequent time points. By analyzing the goodness of fit of these interaction networks (mean cross-correlation between inferred networks and observed networks) across time windows, we observed that the mean cross-correlation across media types increased after the early phase of *in vitro* culture (Fig. S4).

We initially compared ensemble networks at the ASV level across replicate communities cultured in the same media. However, we observed very few interactions were conserved, which is consistent with lower levels of ASV conservation observed between replicate communities. We hypothesized that neutral processes acting during initial community assembly in replicate reactors led to different founder communities of ASVs from the complex fecal sample. However, environmental filtering due to carbohydrate composition could be selected for ASVs with similar functions. To test whether this may be the case, we assessed conserved interactions at the genus level, as conserved functions have been previously observed to occur at the genus level in human fecal communities (54). We found that interactions were conserved between replicates within a medium at the genus level.

**Fig 5:**
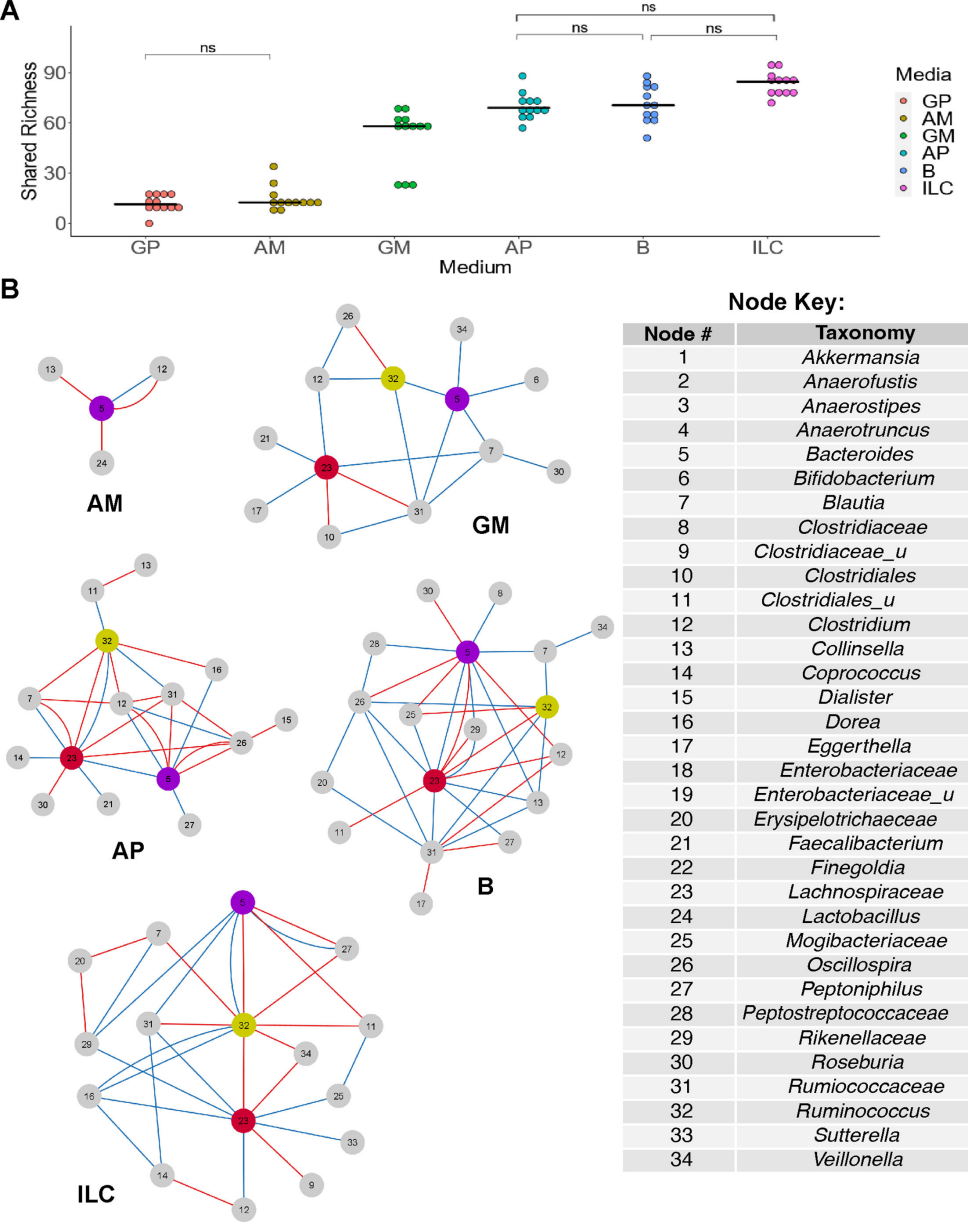
Ensemble networks of conserved interactions demonstrate conserved structure of communities cultured in media that support high shared microbiota richness and within-group similarity. (**A**) Shared microbiota abundance measured across three late culture windows for each FSA replicate culture (*n* = 4), cultured in the media indicated. Unless otherwise noted (ns, for non-significant) comparisons between medium were highly significant with an adjusted *P* value < 5 × 10^−5^ from Tukey’s HSD (**B**) Conserved ensemble networks were built for each media from the networks inferred from the window with the highest quality of fit for each replicate cultured in AM, GM, AP, B, and ILC as marked below each network. A key describing taxa for each numbered node is provided. Positive interactions are indicated by red lines and negative interactions are indicated by blue lines. An ensemble network for GP could not be determined due to a lack of conserved interactions.

Our analysis of the ensemble models primarily focused on FSA samples cultured in B, AP, AM, GP, GM, and ILC (Fig. 5), because the frequency of sample collection in this study facilitated higher-quality ensemble models (data from FSA and FSB in NS, MM, and IL can be found in Fig. S5). Among these ensemble models, we saw a distinct trend in the number of nodes, representing interacting taxonomic groups, and the shared richness of the cultures (Fig. 5A). These networks were not driven solely by correlations between genera, but rather population dynamics fit to generalized Lotka-Volterra models by LIMITS (50), as was demonstrated by different network features between media (e.g., AP, ILC, and B). However, it is important to note that not all taxonomic groups were represented in the networks, as not all groups had conserved (or any) interactions detected in some conditions.

**Fig 6:**
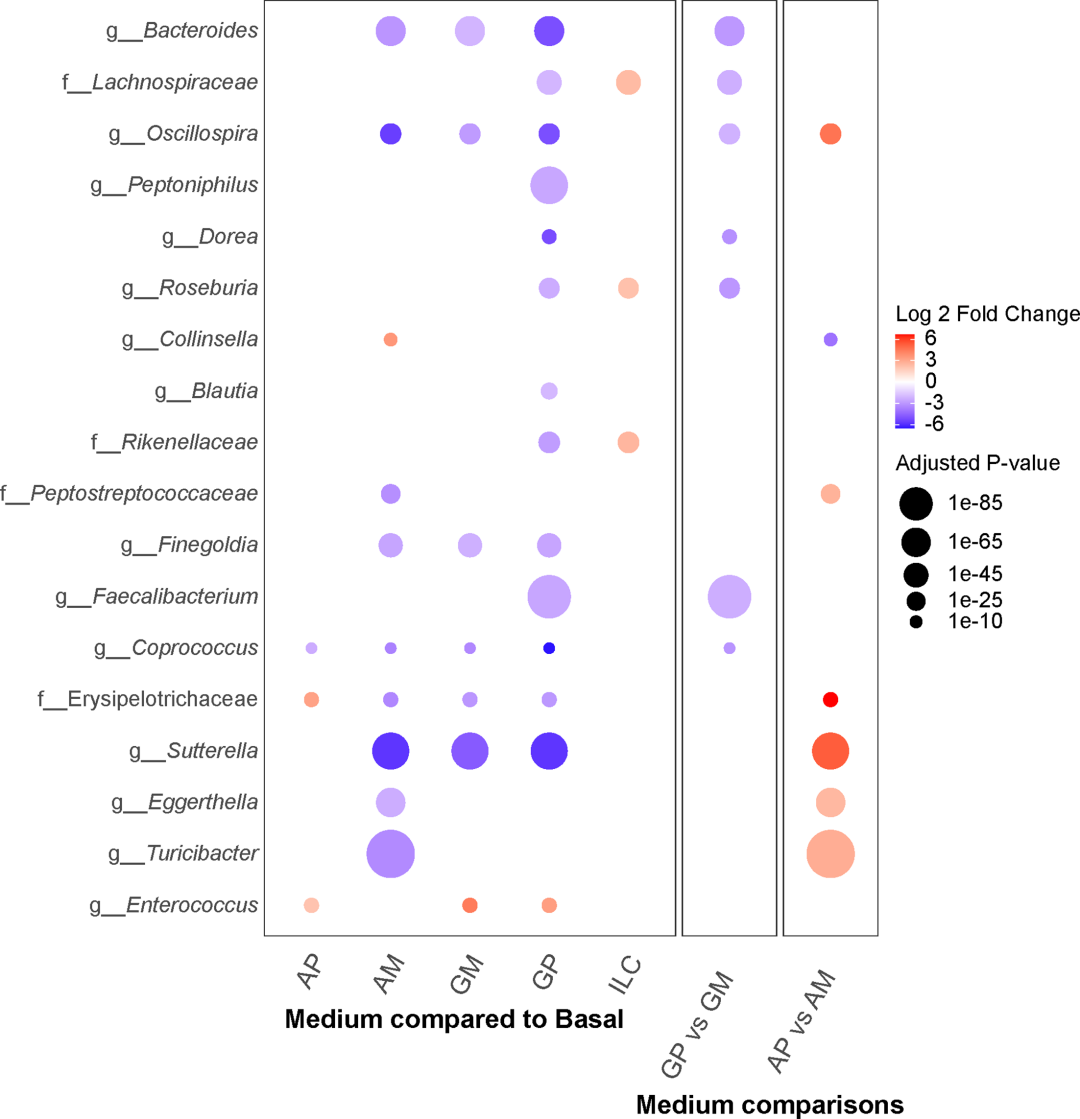
Differential abundance of taxa between FSA communities cultured in different media ASVs were binned into shared taxa at the genus level (or lowest level of taxonomic resolution available for taxa whose classification could not be determined at the genus level). Differential abundance of taxa between communities cultured in different media types was determined using DESeq2. Color indicates the log_2_-fold change in abundance between cultures, and the size of the points indicates the statistical significance of the abundance difference. Unless otherwise indicated, media were compared to medium B as baseline. Comparisons were also made between communities cultured in overrepresented polysaccharide media (GP and AP) and their respective overrepresented monosaccharide media (GM and AM).

From the ensemble models, we found three consistently central taxonomic groups, *Bacteroides*, *Lachnospiraceae*, and *Ruminococcaceae* of unclassified genera (Fig. 5B). Particularly *Lachnospiraceae* and *Ruminococcaceae* of unclassified genera exhibit conserved centrality in media with high CheSL Shannon diversity which produce complex ensemble networks. While the loss of *Lachnospiraceae* spp. interactions in low CheSL Shannon diversity networks could possibly be explained by differences in abundance between media types (e.g., the negative log_2_ fold change in GP compared to B), differences in abundance of *Ruminococcaceae* spp. (and *Lachnospiraceae* spp. in AM medium) could not explain the absence of interactions between communities cultured in different media, as these species were not differentially enriched (Fig. 6). We also found greater connectivity in conserved networks among media types which supported the highest microbiota richness. To further support future uses of ILC medium, we found communities cultured in this medium exhibited more conserved, positive interactions between the three central taxonomic groups mentioned earlier (Fig. 5F). Overall, as we increased CheSL Shannon diversity in our media, we observed more conserved relationships across replicate cultures, suggesting that we may be able to not only increase microbiota richness but also the reproducibility of microbial interactions in cultures derived from complex sources.

For media with low conservation of interactions between replicate networks (i.e., GP and AP), we can gain some insights into the effects of media on community composition through differential enrichment analysis (Fig. 6). We observed that most taxa decreased in abundance in GP compared to B (Fig. 6), except for *Clostridium* spp. and *Bifidobacterium* spp. (Table S3). For AM, we saw a similar selection against many genera, but enrichment of *Collinsella* spp. (Fig. 6). Based on the ensemble network, this enrichment may be driven by *Bacteroides* spp. (Fig. 5B and 6). Alternatively, enrichment may be due to the comparatively low ratio of monosaccharides to polysaccharides, as was previously found in human fecal samples (55).

## DISCUSSION

In this work, we developed novel metrics to describe carbohydrate complexity based on CheSL richness, CheSL Shannon diversity, and CheSL Shannon evenness and demonstrated that mixtures of carbohydrates with higher levels of CheSL Shannon diversity supported growth of human fecal microbial communities with higher richness and conserved microbial interactions. This was contrary to our initial hypothesis that CheSL richness would be the primary driver of microbial richness and points to a role for CheSL Shannon evenness (which also contributes to CheSL Shannon diversity) in microbial community assembly. We hypothesize that CheSL Shannon evenness is most likely to contribute to increased microbiota richness when carbohydrate mixtures have lower levels of CheSL Shannon diversity as a result of high levels of CheSL Shannon unevenness. Specifically, when species that occupy different carbohydrate niches compete for shared resources, the generation of higher levels of biomass by the species that occupy the carbohydrate niche with higher concentrations may deplete other limiting nutrients that will lead to the extinction of other species unable to compete for this limiting nutrient. Thus, we believe these observations do not contradict, but rather build upon nutrient-niche theory (23, 24) and integrate the concepts of competitive fitness in the context of community stability and carbohydrate diversity.

This work builds on the body of emerging literature linking carbohydrate complexity to microbial community composition and function. Similar to what was observed by Chung et al. and Yao et al., media with higher levels of CheSL Shannon diversity support higher levels of microbiota richness in communities cultured from fecal samples across the samples tested (26, 27). As Ostrem Loss et al. used a defined community of 10 microbes selected to provide the representation of microbiota diversity, they did not see the effects of carbohydrate complexity on richness (29). However, reanalysis of their data demonstrated correlations between microbiota diversity and carbohydrate complexity (Fig. S6). We also observe the correlation between CheSL Shannon diversity and microbial diversity (Fig. S6), but the correlations observed with microbiota richness were of primary interest for this study. Of note, the CheSL Shannon evenness metric that we describe here is very similar to the carbohydrate complexity measure determined by Loss and colleagues, with the exception that we calculate a single fractional proportion of monosaccharides and linkages, whereas Loss et al. sum the Shannon diversity of fractional monosaccharides and Shannon diversity of fractional linkages which are calculated separately (29). Our work further expands on the studies of Ostrem Loss et al. through the development of the CheSL Shannon diversity, which for the data analyzed in this study exhibits stronger correlation with microbiota richness than CheSL Shannon evenness.

While the trends we observed are consistent with the data cited above, other studies have found more complex relationships between carbohydrate complexity and microbiota richness that are likely due to differences in the ability of microbial communities to cleave linkages identified (28, 56, 57). Degradation of complex polysaccharides is dependent upon the presence of specific carbohydrate-active enzymes (CAZymes) within microbial community members (58). In the absence of enzymes able to break unique linkages within complex polysaccharides, monosaccharides remain inaccessible and cannot provide unique niches. The presence of specific CAZymes which allow degradation of linkages are the gateway to energy for the microbes, and in turn, linkages serve as a representation of the latent energy granting niche space. Thus, it was not entirely surprising when we observed that when more carbohydrate mixtures and microbial communities were analyzed, correlations between CheSL diversity and microbiota richness were lower (Fig. S2). However, we suggest that the predictive accuracy of CheSL can be improved with prior knowledge of the CAZyme diversity within the complex community being targeted. Inherent assumptions used in this study were that the complex fecal microbiota would be capable of utilizing the selected carbohydrates as they are abundant in diet or are host-derived. One should be careful in the use of CheSL for predictions in cases of low initial functional diversity or addition of uncommon substrates to communities that likely lack the enzymatic potential for degradation (e.g., cellulose to human fecal sample-derived community). Further, the CheSL metrics should be applied across media which are controlled with respect to other substrates. Finally, it is also important to note that CheSL measures are estimates based on published literature and will improve as additional carbohydrate composition and linkage information is published.

From our observations, among samples cultured in media with the greatest CheSL Shannon diversity (B, AP, and ILC), there is higher similarity between microbial interaction network structure and shared microbiota richness. This may suggest the diversity of nutrient niches inferred by the abundance and evenness of linkages may control the reproducibility of community assembly from complex microbial samples in continuous flow bioreactors, thereby impacting the shared microbiota richness of the samples. Ostrem Loss et al. similarly observed that higher carbohydrate complexity led to higher reproducibility in community assembly (29). From our findings, the initial assembly of a community around *Bacteroides*, *Lachnospriaceae*, and *Ruminococcaceae* may result in a network of interactions supporting reproducible cultures with greater microbiota diversity under these culture conditions.

We also found enrichment and microbial associations previously observed in *in vivo* systems exposed to similar treatments—another indicator of our *in vitro* system’s capability to support representative communities. Particularly the relationship between *Bacteroides* and *Lachnospiraceae* was supported through studies demonstrating cross-feeding between *B. thetaiotaomicron* and *Anaerostipes caccae* (59). *Clostridium sensu stricto* and *Bifidobacterium* have previously been observed in humans consuming diets high in starch, aligning with our GP medium—which over-represents soluble starch for which these cultures continued to support these taxa despite significant reduction in diversity (31, 60, 61).

Communities formed in conditions with high CheSL Shannon diversity also exhibited enrichment of genes for potential Stickland fermentation of amino acids. Consistent with these observations, we observed higher levels of peptide depletion in these media. Future studies will be needed to determine whether the utilization of peptides in media with high CheSL Shannon Diversity is conserved across other microbial communities and media types. Typically, proteolytic fermentation is associated with negative health outcomes, with a tradeoff between saccharolytic and proteolytic microbes (62). In our studies, peptide consumption occurred in the presence of dietary fibers and in communities enriched with genes for butyrate production, suggesting that potential beneficial functions of the microbiota were also maintained.

Like any ecological set of interactions, interactions between GI microbiota are directly determined by the biotic and abiotic properties of the environment to which the community is exposed. Using approaches that adhere to limitations in data analysis and modeling as described in this work, we gained additional resolution into the relationships between fecal bacteria and the potential interaction landscapes which can be realized from the availability of specific substrates. From progression in process-based approaches to interaction prediction, we further informed and connected population dynamics with metabolic potential and ecological theories of nutrient niche partitioning (23, 24). Using CheSL Shannon diversity as a guide when designing *in vitro* culture media has the potential to improve reproducibility and increase the similarity of *in vitro* communities to those found in the fecal samples without introducing unknown dietary components that would be found in media supplements, such as ileostomy effluents or foods that have undergone simulated digestion. Our initial test at an improved carbohydrate medium supports this potential, with ILC granting a similar network structure to IL and the greatest shared richness of media which had a known substrate concentration (IL being of complex origin). By improving the reproducibility of community assembly in continuous flow conditions, we can better detect differences between samples and reactors driven by other interventions. Additionally, the experimental conditions we used, the improved quality of fit realized using a continuous flow bioreactor, strict filtering, and division of our time series into windows which represent transitory states in the community, all together may aid in inferring interactions for future mechanistic investigation of microbial metabolic interactions.

## MATERIALS AND METHODS

### Samples used in this study

Fecal samples were previously collected from two adult volunteers who were self-described as healthy and had not consumed antibiotics within the previous 2 months (30). Samples were homogenized under anaerobic conditions, aliquoted into anaerobic tubes, and stored at −80°C until cultivation. Ileostomy effluents were collected from participants who had ostomies placed due to previous illness or injury as previously described (63). Samples were stored at −20°C until use. Informed consent was obtained prior to sample collection according to a protocol approved by the Institutional Review Board of Michigan State University (10-736SM).

### Media used in this study

Microbes from fecal samples were cultured in bioreactor basal medium that varied in carbohydrate composition. 1 L of basal medium contained 1 g tryptone, 2 g proteose peptone #3, 2 g yeast extract, 0.4 g sodium chloride, 0.5 g bovine bile, 0.01 g magnesium sulfate, 0.01 g calcium chloride, and 2 mL Tween 80, which was sterilized by autoclaving. 2 g sodium bicarbonate, 0.2 g inulin from chicory, 0.1 g arabinogalactan from larch wood, 0.2 g soluble starch, 0.04 g potassium phosphate dibasic, 0.04 g potassium phosphate monobasic, and 0.001 g vitamin K3 were filter sterilized and added to each liter of basal medium after autoclaving. Different variations of carbohydrates added to basal medium were used to test the impact of carbohydrate composition and concentration on microbial communities (Table S1). NS (No starch) lacked soluble starch and contained 0.15 g/L d-cellobiose, 0.15 g/L maltose, and 0.04 g/L d-glucose in addition to basal medium. MM (mucosal monosaccharides) contained an additional 0.2 g/L of soluble starch, 0.05 g/L N-acetylglucosamine, 0.05 g/L N-acetylneuraminic acid, 0.05 g/L d-glucuronic acid, and 0.05 g/L l-fucose. 5MM (5× higher mucosal monosaccharides) contained an additional 1.8 g/L soluble starch (2 g/L final concentration), 0.2 g/L N-acetylglucosamine, 0.2 g/L N-acetylneuraminic acid, 0.2 g/L d-glucuronic acid, and 0.2 g/L l-fucose (0.25 g/L final concentration each). GP (glucose polysaccharide) contained an additional 1.8 g/L starch (2 g/L final concentration). GM (glucose monomer) contained an additional 1.96 g/L d-glucose (2 g/L final concentration). AP (arabinogalactan polymer) contained an additional 1.9 g/L of arabinogalactan (2 g/L final concentration). AM (arabinogalactan monomers) lacked arabinogalactan and instead contained the two primary monosaccharides present in arabinogalactan (34, 64)—1.79 g/L galactose and 0.21 g/L arabinose. IL (ileostomy effluent) contained an additional 0.2 g/L soluble starch, 0.05 g/L N-acetylglucosamine, 0.05 g/L N-acetylneuraminic acid, 0.05 g/L d-glucuronic acid, 0.05 g/L l-fucose, and 100 mL of ileostomy effluent. Ileostomy effluent was thawed, pooled from multiple donors, and filtered through a coffee filter to remove particulates prior to sterilization along with base medium by autoclaving. Carbohydrate diversity of ileostomy effluent was estimated from references (44, 45). Finally, ILC (ileostomy carbohydrates) reduced the concentrations of arabinogalactan, inulin, and soluble starch to 0.05 g/L for each of these carbohydrates and also contained 0.05 g/L cellobiose, 0.05 g/L fructose, 0.05 g/L galactose, 0.05 g/L N-acetylneuraminic acid, 0.05 g/L N-acetylglucosamine, 0.05 g/L l-fucose, and 0.05 g/L d-glucuronic acid.

### Culturing human GI microbes in continuous-flow bioreactors

We used MBRAs (30), continuous-flow culture vessels that simulate environmental parameters of the lumen of the distal human colon and maintain communities in steady state based on the carrying capacity of the reactors, to culture communities of human GI microbes from fecal samples. MBRAs are parallelized; therefore, we could culture replicate communities of human GI microbes in medium that varied by carbohydrate composition and concentration and determine how these changes impacted microbial interactions. Prior to inoculation into MBRAs, fecal samples were thawed and homogenized in anaerobic phosphate-buffered saline (PBS) as a 20% wt/vol slurry as previously described (30). In the first experiment, FSA was cultured in NS (*n* = 2 replicates), MM (*n* = 3 replicates), or IL (*n* = 3 replicates) and FSB was cultured in NS (*n* = 3 replicates), MM (*n* = 3 replicates), 5MM (*n* = 3 replicates), or IL (*n* = 3 replicates). Media contamination prevented the cultivation of FSA in 5 MM, whereas technical difficulties led to the loss of third replicate of FSA in NS. Both FSA and FSB, formerly referred to as Fecal Sample B and Fecal Sample C, respectively, were previously characterized when cultured in BRM2, a medium highly similar to NS, with an 8-h retention time (30). For these experiments, 16 h after inoculation, continuous flow was initiated at 0.94 mL/h (16 h retention time for 15 mL minibioreactor). About 1 mL samples was collected from each culture daily for 10 days as previously described (30).

In the second experiment, FSA was selected for studies because of fecal sample availability. FSA was cultured in B, GP, GM, AP, AM, and ILC media, with *n* = 4 replicates/medium. As before, continuous flow was initiated 16 h after inoculation at 0.94 mL/h. About 1 mL samples was collected every 12 h for 8 days. The increased frequency of sampling and number of replicates were to improve our ability to subsequently infer microbial interactions from data. In both experiments, MBRAs were maintained in an anaerobic chamber (Coy Laboratories) heated to 37°C with a gas atmosphere of 90% N_2_, 5% CO_2_, and 5% H_2_.

### 16S rRNA gene amplicon sequencing

The V4 region of the 16S rRNA gene was amplified directly from mechanically disrupted cells with broad-range 16S rRNA primers 515F/805R as previously described (30). Purified amplicons were pooled in equimolar concentration and sequenced using Illumina MiSeq v2 according to the manufacturer’s protocol. Sequence data were processed using mothur V1.35.0. Sequences were aligned using the Silva r132 16S rRNA gene sequence database (65). Any chimeric sequences were removed following identification by UCHIME, and any contaminating sequences identified as chloroplasts, mitochondria, Archaea, or Eukarya were removed as well (66). Sequences were denoised using mothur’s pre.cluster function, giving ASVs, and taxonomy was assigned using the Silva r138 16S rRNA gene sequence database. Downstream analysis of the taxonomic and count data were handled in R, using the phyloseq package (67, 68). Code for analysis is published at https://github.com/HughMcCullough/CheSL, along with an ASV table.

### Data pre-processing for computational network inference

Since Lotka-Volterra equations model niche-based effects determining population dynamics, multiple filtering steps were required to mitigate spurious interactions. During the early phases of culture, two factors could influence community stability: the loss of ASV presence as non-viable cells were removed by dilution and partnership switching as communities adapted to the culture conditions. To improve LIMITS inferred model quality of fit, we tested whether a sliding window approach, sampling time regions across the time series, could improve the consistency of interactions over time. We tested windows of varying sizes from 6 to 15 time points, across the entire time series. To inhibit performance loss from transient ASVs, we filtered ASVs by minimum presence across all time points within a window, requiring the presence of a single read from an ASV across all time points. This pre-processing avoided issues associated with taxa falling and rising above the threshold of detection, which would influence the model similarly to migration and extinction events and disturb inferences of a fixed ecosystem’s interactions (69). From this filtering, we obtained what we call a “shared microbiota richness,” which represents the count of ASVs found consistently across time, excluding ASVs that may be alternating above and below the threshold of detection.

### Inference of interspecies interactions using LIMITS

LIMITS analysis was performed using the seqtime package in R by Faust et al. (51). Prior to running LIMITS, we preprocessed the data as described above. From there, we applied the LIMITS algorithm using a sliding window approach as described above. Inferred interactions were then used to simulate time series for the given window of time, and the resulting simulated data were compared to the experimental data. Mean cross-correlations were used to quantify the quality of fit of the simulated data to the experimental data. Specifically, mean cross-correlations were calculated by determining the correlation between each taxon’s simulated and experimental data over the time series; the mean correlation of all taxa was then calculated; this method of quality of fit analysis was also implemented through the seqtime package (51). This sliding window method granted greater quality of fit than applying LIMITS to the entire experiment’s time series. In addition to improved quality of fit, a sliding window approach allowed us to select the time windows which most likely represent interactions driven by deterministic effects, rather than including data from the first day(s) of cultivation which likely included influences of founder effects, partner selection, and adjustment to the bioreactor system.

From each replicate and window, a network was inferred using LIMITS. To identify conserved interactions, we calculated consensus across networks from replicates of the same fecal sample and media culture conditions using the same fecal sample inoculum. After calculating average interaction values and filtering by a consensus threshold of 2, meaning at least two networks identified the interaction with the same directionality, we obtained ensemble networks for each media type.

### Characterization of chemical subunit and linkage diversity

As described in the results, we developed CheSL richness, CheSL Shannon diversity, and CheSL Shannon evenness measures to calculate the complexity of carbohydrate mixtures based on distinct linkages within di- and polysaccharides or the presence of unique monosaccharides. For example, the polysaccharide arabinogalactan is primarily composed of arabinose (80%) and galactose (15%) monomers (34) that are linked in eight unique types of linkages within the polysaccharide (Table 1), with the relative abundances of these linkages also known. We additionally informed the ratios of our linkages for amylopectin from literature, which indicated predominantly α-1-4 linkages, with 5% of linkages being α-1-6 linkages (35). For disaccharides, which have a single linkage type, there is no effective change in diversity when applying CheSL if it is the sole carbohydrate, but it is important to decompose into linkage types in cases where there is overlap. For example, we find that maltose, with α-1-4 linkages, contributes to the same pool as amylopectin (37). While cellobiose and inulin have their own unique linkage pools—β (1-4) glucose for cellobiose and β(2-1) fructose and α-(l-2) glucose for inulin (27, 36). We estimated fructan linkage diversity from a study containing glycosidic linkage proportions from wheat kernels, yielding six distinct linkage types (38). For estimations of linkage diversity for CheSL calculations to apply to Chung et al.’s data, we referenced literature for pectin, beta-glucan, and glucomannan, respectively (46–48). Composition and linkage data not described here can be found in Table 1 and Supplementary Tables S1 and S2. Figure 2 provides a representation of how CheSL diversity can be calculated across a medium containing multiple carbohydrates.

### Statistical analysis—comparison of means and correlations

To make comparisons for media shared richness, we used the R package MASS’s implementation of a negative binomial GLM, glm.nb, as the richness did not fit a normal distribution determined by the Shapiro-Wilk test, and the data were over-dispersed for a Poisson distribution (70). Pairwise comparisons were made with the R package emmeans, and Tukey’s HSD was used to adjust for family-wise error rate (71). For correlations, the R package ggpubr was used alongside tidyverse and ggplot2 to visualize and calculate the Pearson correlation between diversity metrics (72–74).

### Inferred metabolic functional potential with PICRUSt

After characterization of conserved interactions and visualization using Cytoscape, we inferred Enzyme Commission Pathway abundance for our cultures from fully sequenced isolates with similar 16S rRNA gene sequences using PICRUSt2 (75). The potential significance of Pfam pathway abundance was assessed using STAMP (76).

### Measurement of amylose/amylopectin ratio

To measure the ratio of amylose/amylopectin for this study, we use a NEOGEN Megazyme amylose/amylopectin Assay kit, product code K-AMYL. The assay was performed according to the manufacturer’s instructions. This kit was used to determine the proportions of amylose and amylopectin comprising the soluble starch used in various media formulation. This ratio was then used to calculate CheSL diversity measures.

### Differential analysis of microbial abundances

We performed differential abundance analysis with DESeq2 (77) between communities cultured in basal bioreactor medium (B) and media with differing carbohydrate composition. The DESeq2 model we used to explain composition as a function of media type, with time as a covariate. Additionally, we performed DESeq2 analysis between communities cultured in media composed of the polymer form (starch [GP] and arabinogalactan [AP]) and respective monomer forms (glucose [GM] and galactose and arabinose [AM]), as they represent potential differences between monosaccharide-based niches and linkage-based niches at the genus level. Changes in abundance that were identified by DESeq2 as significant and had an absolute fold-change in abundance greater than 2 were plotted.

## Data Availability

16S rRNA gene data are available in the NCBI Sequence Read Archive under BioProject ID PRJNA1146550. Code and input files for analysis are published at https://github.com/HughMcCullough/CheSL.

## SUPPLEMENTAL MATERIAL

Supplemental figures spectrum.02618-24-s0001.pdf Fig. S1 to S6.

Supplemental tables spectrum.02618-24-s0002.xlsx Tables S1 and S2.

ASM does not own the copyrights to Supplemental Material that may be linked to, or accessed through, an article. The authors have granted ASM a non-exclusive, world-wide license to publish the Supplemental Material files. Please contact the corresponding author directly for reuse.
