# Erratum for McCullough et al., “Diversity in chemical subunits and linkages: a key molecular determinant of microbial richness, microbiota interactions, and substrate utilization”

**DOI:** 10.1128/spectrum.02435-25

**Published:** 2025-09-09

**Authors:** Hugh C. McCullough, Hyun-Seob Song, Jennifer M. Auchtung

## ERRATUM

Volume 13, no. 4, e02618-24, 2025, https://doi.org/10.1128/spectrum.02618-24. Page 4, Table 1: Rows 9-11, 13, 15, and 22 of the Linkages column should read “α-(1-4),” “α-(1-4),” “α-(1-6),” “α-(1-2),” “α-(1-4),” and “Glc (1-6)p,” respectively. These errors accumulated due to the visual similarity of the number 1 and the lowercase letter l in some sans serif fonts and copying of the formatting during table generation.

Page 6: The second example equation labeled “(10)” should be “(11).”

