# Supplementary material for: Diversity in chemical subunits and linkages: a key molecular determinant of microbial richness, microbiota interactions, and substrate utilization
Source: Microbiol Spectr. 2025 Mar 6;13(4):e02618-24. doi: 10.1128/spectrum.02618-24 (PMC11970232; doi:10.1128/spectrum.02618-24)
Supplement: Supplemental figures — Fig. S1 to S6. [file spectrum.02618-24-s0001.pdf]

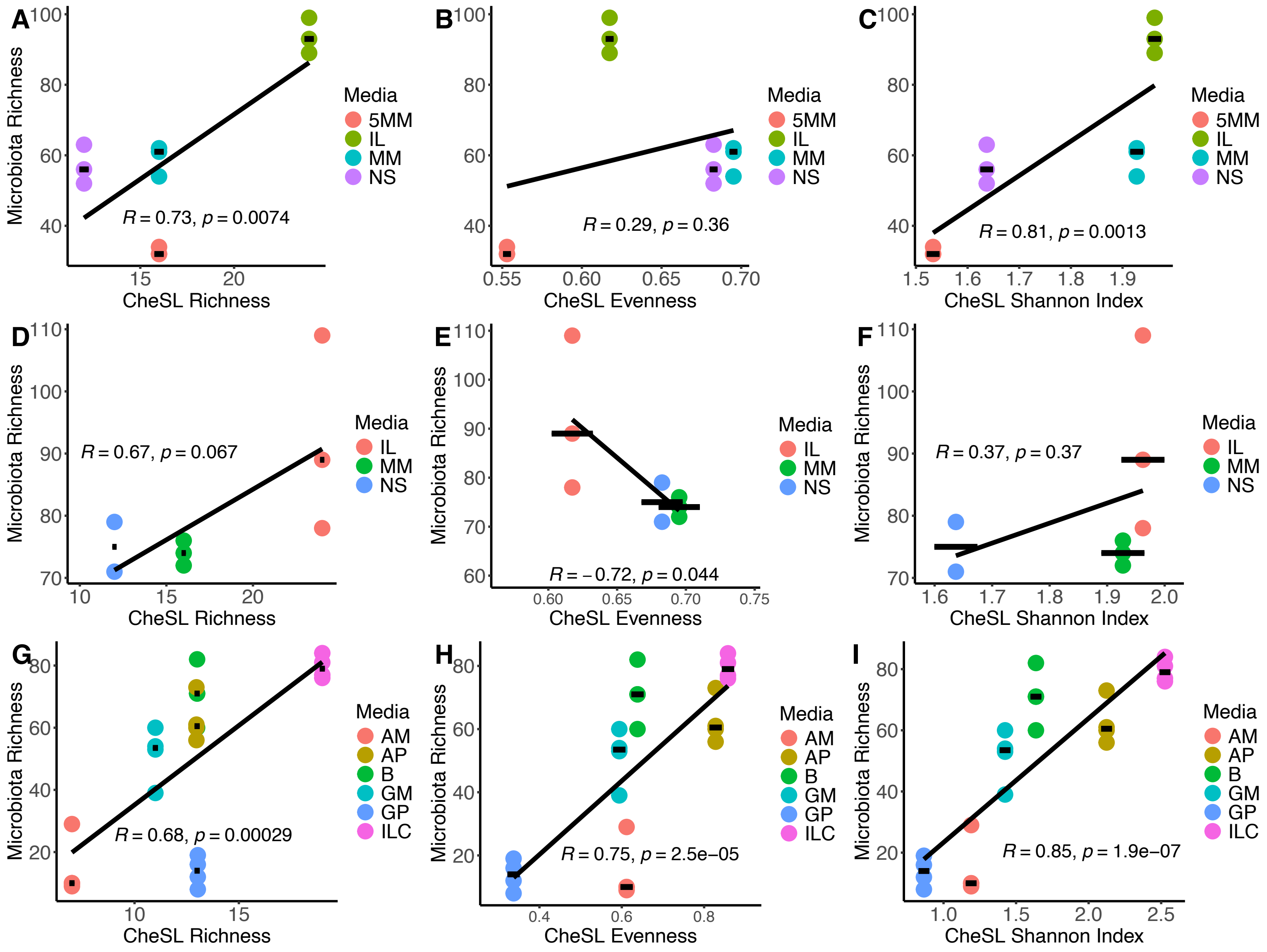

Supplementary Figure S1. Effects of CheSL richness, CheSL Shannon evenness, and CheSL Shannon diversity on microbiota richness within fecal sample communities and between experiments. Correlations between shared ASV richness and (A,D,G) CheSL Richness, (B,E,H) CheSL Shannon evenness, and (C,F,I) CheSL Shannon diversity are shown FSB (A-C) and FSA (D-F) cultured in NS, IL, MM and 5MM (A-C only), and for FSA cultured in B, AM, AP, GM, GP, and ILC (G-I). Pearson correlation coefficients and p-values are reported.

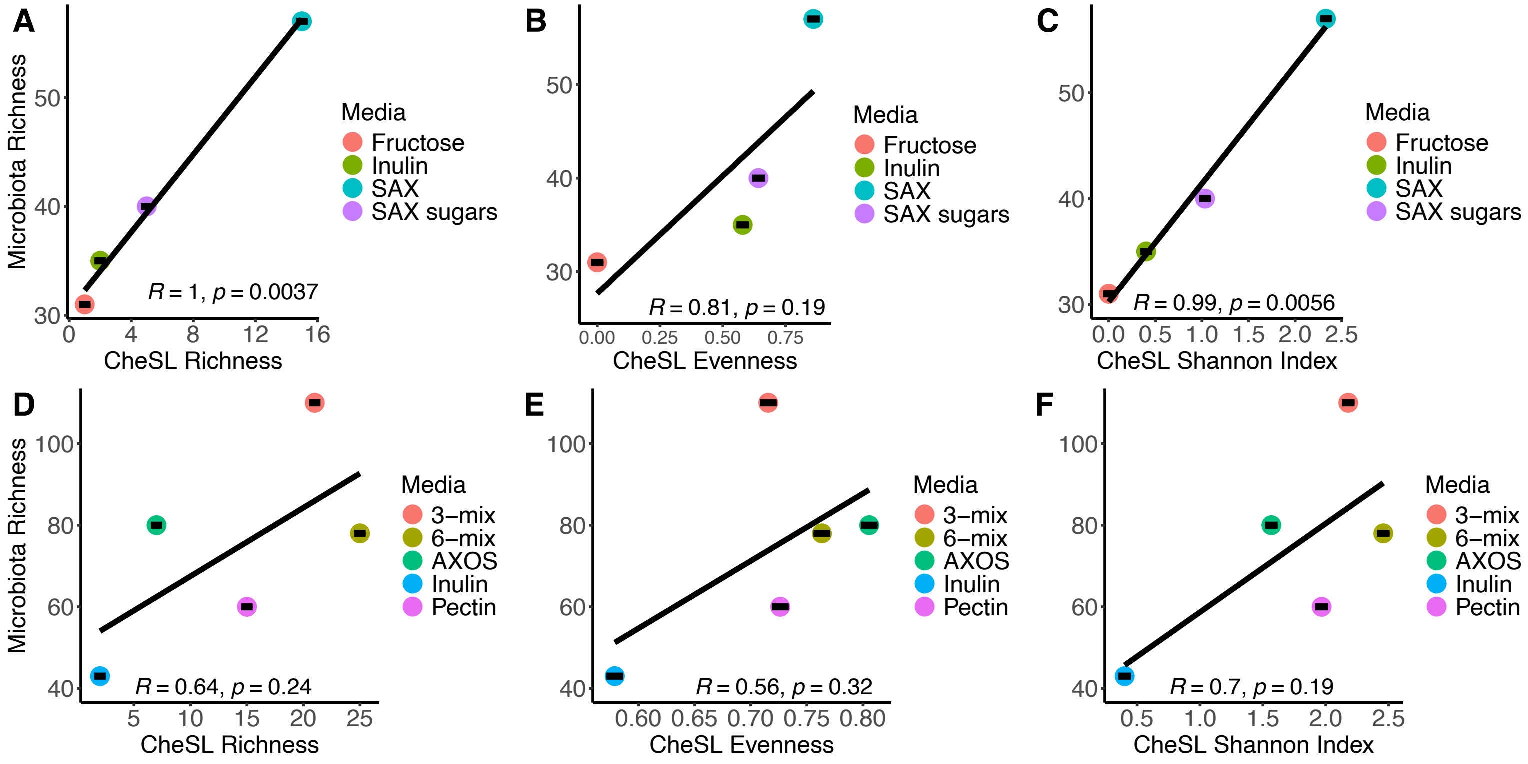

Supplementary Figure S2. Effects of CheSL richness, CheSL Shannon evenness, and CheSL Shannon diversity on microbiota richness from previously collected data. (A-C) Correlations between shared OTU Richness and (A) CheSL richness, (B) CheSL Shannon evenness, and (C) CheSL Shannon diversity from data reported by Yao and colleagues (27). (D-F) Correlations between shared OTU richness on days 15, 18, and 20 for Donor 1 (calculated from data published as Supplementary Table S1) and (D) CheSL richness, (E) CheSL Shannon evenness, and (F) CheSL Shannon diversity from data reported by Chung and colleagues (26). Supplementary Table S2 provide details for CheSL calculations. Pearson correlation coefficients and p-values are reported.

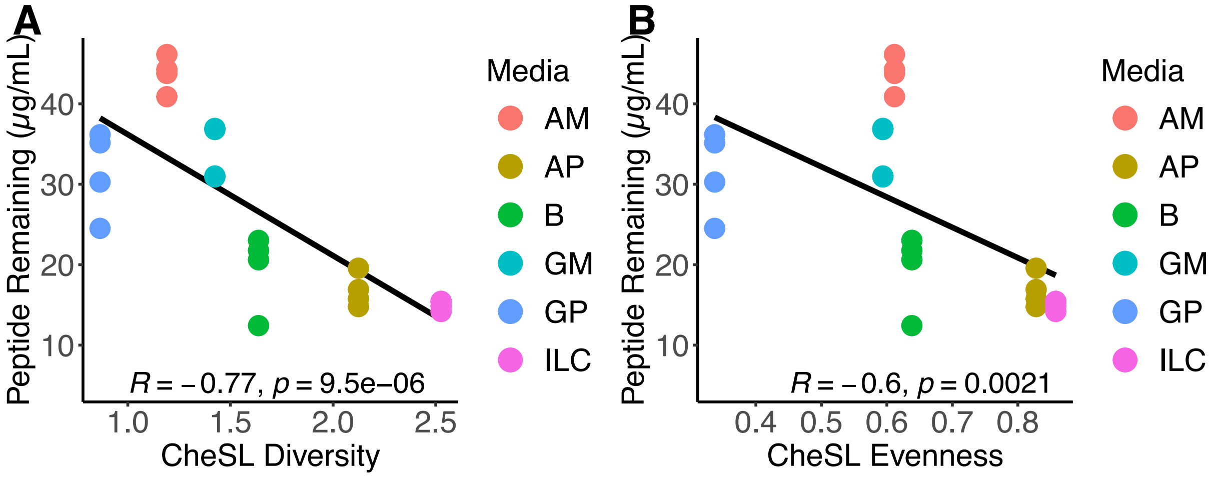

Supplementary Figure S3. Reduction in peptide levels strongly correlates with microbiota richness. As described in Figure 4, we measured peptide levels in supernatants from stable FSA communities cultured in AM, AP, GM, GP, B, and ILC (n=4/medium). Here we report correlations with (A) shared ASV richness and (B) CheSL Shannon evenness. Pearson correlation coefficients and p-values are reported.

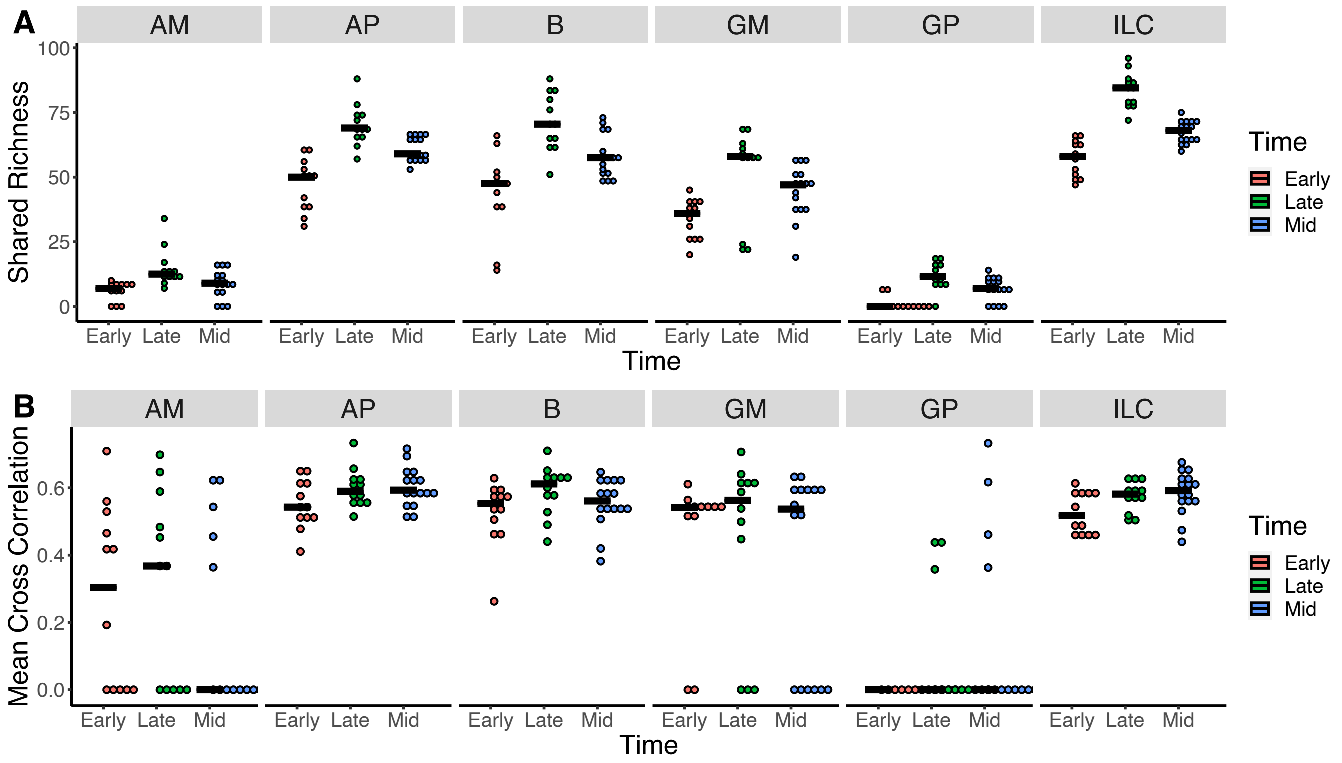

Supplementary Figure S4. Shared richness and mean cross correlation values of FSA communities cultured in AM, AP, B, GM, GP, and ILC across different time points in culture. As described in Methods, we calculated (A) shared richness and (B) mean cross correlation values for three groupings of time windows during early culture (including the first 3 timepoints A-B), mid (excluding the first three and last three timepoints C-D), and late (excluding the first six timepoints E-F) to identify best time windows for building conserved ensemble networks.

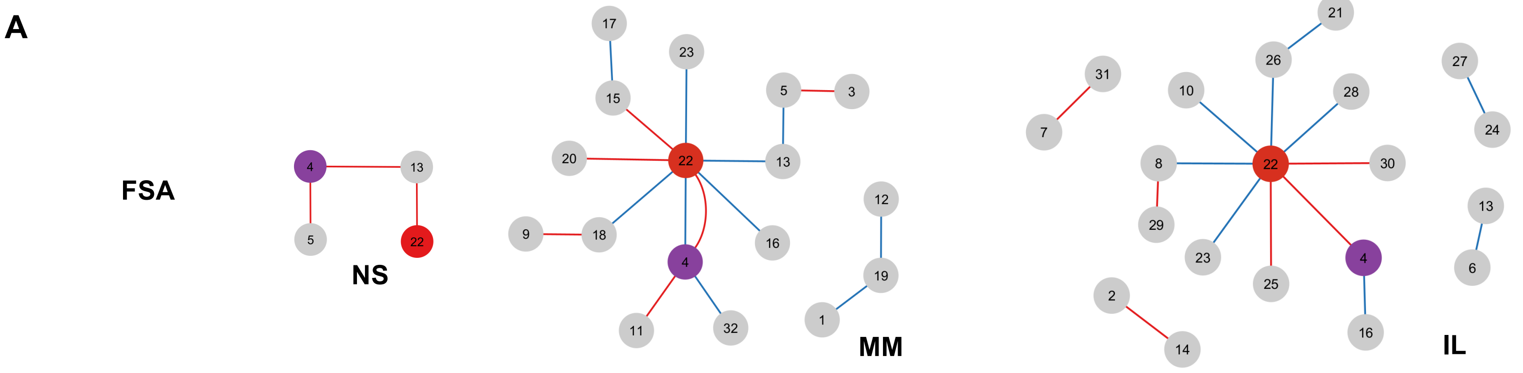

Node #	Taxonomy	Node #	Taxonomy	Node #	Taxonomy	Node #	Taxonomy
**1**	*Alistipes*	**9**	*Coprococcus*	**17**	*Halomonas*	**25**	*Oscillospira*
**2**	*Anaerostipes*	**10**	*Eggerthella*	**18**	*Holdemania*	**26**	*Oscillospiraceae*
**3**	*Anaerovoracaceae_u*	**11**	*Eisenbergiella*	**19**	*Hungatella*	**27**	*Oscillospirales*
**4**	*Bacteroides*	**12**	*Escherichia-Shigella*	**20**	*Incertae_Sedis*	**28**	*Phascolarctobacterium*
**5**	*Blautia*	**13**	*Family_XIII_AD3011*	**21**	*Lachnoclostridium*	**29**	*Phocea*
**6**	*Christensenellaceae_R-7*	**14**	*Firmicutes*	**22**	*Lachnospiraceae*	**30**	*Ruminococcaceae*
**7**	*Clostridia_vadinBB60*	**15**	*Flavonifractor*	**23**	*NK4A214_group*	**31**	*Tissierella*
**8**	*Colidextribacter*	**16**	*GCA-900066755*	**24**	*Oscillibacter*	**32**	*UCG-005*

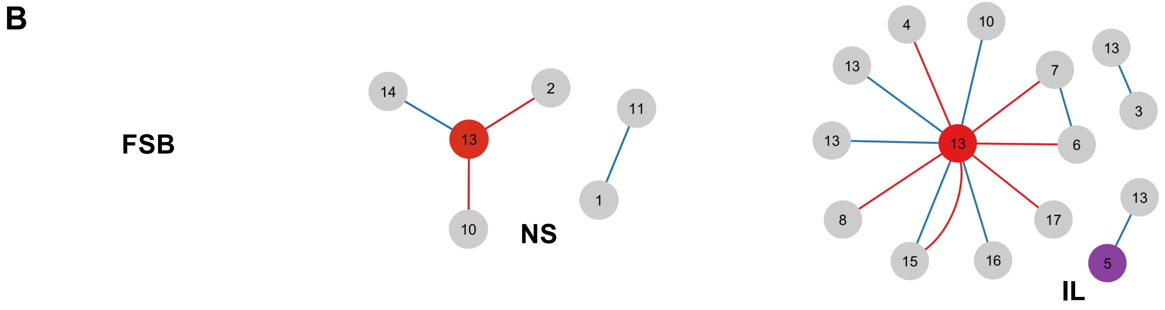

Node #	Taxonomy	Node #	Taxonomy	Node #	Taxonomy	Node #	Taxonomy
1	*Acetanaerobacterium*	6	*Blautia*	10	*Family_XIII_AD3011*	14	*Oscillospiraceae*
2	*Akkermansia*	7	*Butyricicoccus*	11	*Lachnoclostridium*	15	*Ruminococcaceae*
3	*Alistipes*	8	*Christensenellaceae_R-7*	12	*Lachnospiraceae*	16	*Tissierella*
4	*Anaerovoracaceae_*	9	*Erysipelotrichaceae*	13	*Oscillibacter*	17	*UBA1819*
5	*Bacteroides*						

Supplementary Figure S5. Conserved ensemble networks for FSA and FSB cultured in NS, MM, and IL. Conserved ensemble networks were built for each media/fecal sample combination from the networks inferred from the window with the highest quality of fit for each replicate. A key describing taxa for each numbered node is provided. Positive interactions are indicated by red lines and negative interactions are indicated by blue lines. (A) Networks for FSA communities grown in NS, MM, or IL as marked below each network. (B) Networks for FSB communities grown in NS and IL as marked below each network. Ensemble networks for FSB grown in MM and 5MM could not be determined due to lack of conserved interactions.

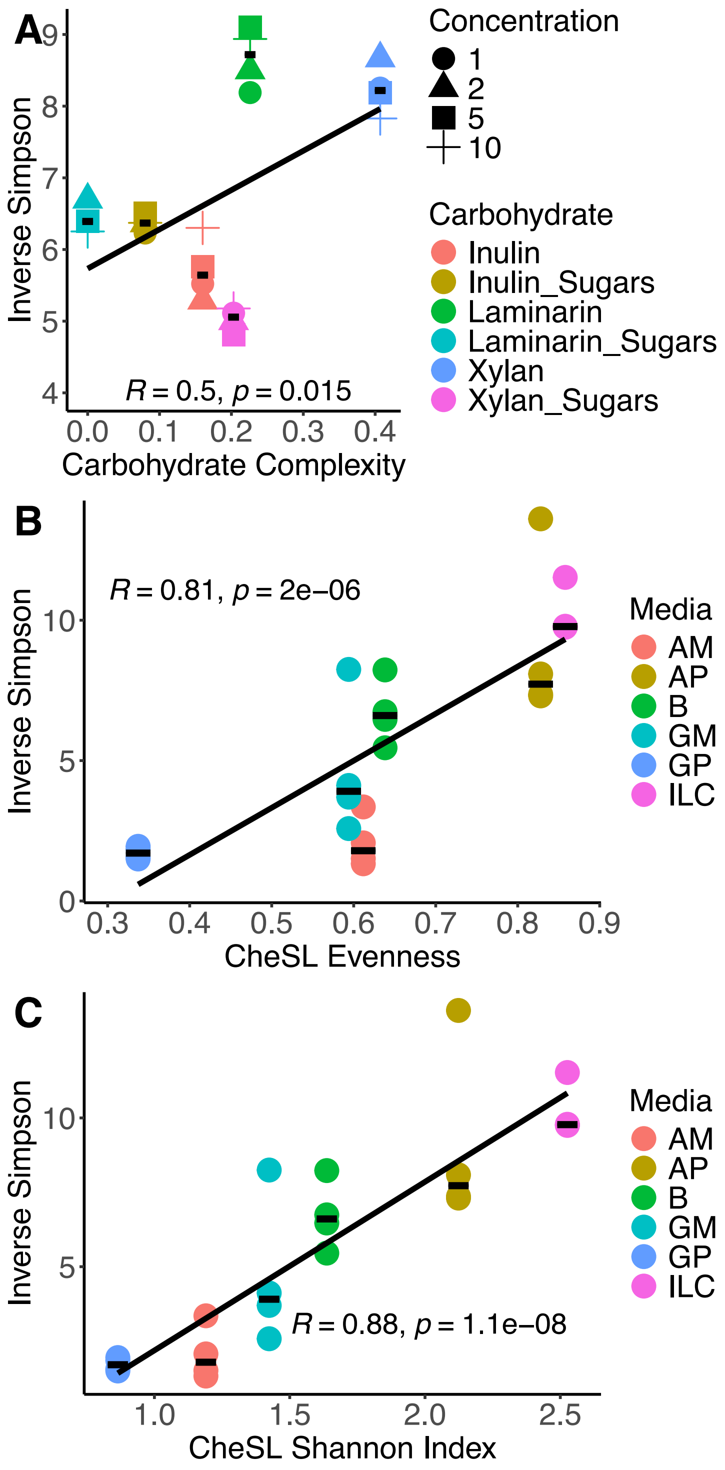

Supplementary Figure S6. Correlation between carbohydrate diversity and Inverse Simpson diversity for data described by Loss and colleagues. (A) Fractional data for microbe abundance provided in previously published Supplementary Table 2 was converted to whole numbers and used to calculate Inverse Simpson microbial diversity with mothur v.1.48. Inverse Simpson microbial diversity values were plotted as a function of the carbohydrate diversity measure reported by Loss. Pearson correlation coefficient and p-value are reported. Panels (B) and (C) repeat data from Figure S1 for reference and show correlations between ASV abundance for FSA communities cultured in the indicated media and (B) CheSL Shannon evennes and (C) CheSL Shannon diversity.
